# A Tripartite Synapse Model in *Drosophila*

**DOI:** 10.1371/journal.pone.0017131

**Published:** 2011-02-16

**Authors:** Rie Danjo, Fumiko Kawasaki, Richard W. Ordway

**Affiliations:** Department of Biology and Center for Molecular and Cellular Neuroscience, Pennsylvania State University, University Park, Pennsylvania; University of Missouri, United States of America

## Abstract

Tripartite (three-part) synapses are defined by physical and functional interactions of glia with pre- and post-synaptic elements. Although tripartite synapses are thought to be of widespread importance in neurological health and disease, we are only beginning to develop an understanding of glial contributions to synaptic function. In contrast to studies of neuronal mechanisms, a significant limitation has been the lack of an invertebrate genetic model system in which conserved mechanisms of tripartite synapse function may be examined through large-scale application of forward genetics and genome-wide genetic tools. Here we report a *Drosophila* tripartite synapse model which exhibits morphological and functional properties similar to those of mammalian synapses, including glial regulation of extracellular glutamate, synaptically-induced glial calcium transients and glial coupling of synapses with tracheal structures mediating gas exchange. In combination with classical and cell-type specific genetic approaches in *Drosophila*, this model is expected to provide new insights into the molecular and cellular mechanisms of tripartite synapse function.

## Introduction

Previous studies have revealed important roles for glia in synapse development and function as well as neurological disorders including Amyotropic Lateral Sclerosis (ALS), Epilepsy, Huntington's Disease and drug abuse (reviewed in [1]–[9]). Despite this progress, new and complementary approaches are needed to further define the molecular mechanisms of tripartite synapse function. Genetic analysis in *Drosophila* has been of central importance in studies of neural development and function, including extensive work on glial biology (reviewed in [10]–[13]). However, with respect to tripartite synapses, the widely used embryonic and larval neuromuscular synapse preparations do not exhibit direct synapse-glia interactions [14], [15] and the general existence of tripartite synapses in *Drosophila* has remained unclear (thoughtfully reviewed in [13]). The present study builds on our development of the adult Dorsal Longitudinal Muscle (DLM) neuromuscular synapse model for analysis of molecular mechanisms determining conserved functional properties of glutamatergic synapses (c.f. [16]). Notably, Birman and colleagues have shown that these synapses are closely associated with glia and that glial glutamate transport by the single *Drosophila* EAAT-type glutamate transporter contributes to synaptic function [17]–[21]. We have now extended our studies of adult DLM neuromuscular synapses to establish a tripartite synapse model exhibiting conserved morphological and functional properties with respect to mammalian tripartite synapses.

## Results

### Tripartite morphology of adult DLM neuromuscular synapses

**Figure 1:**
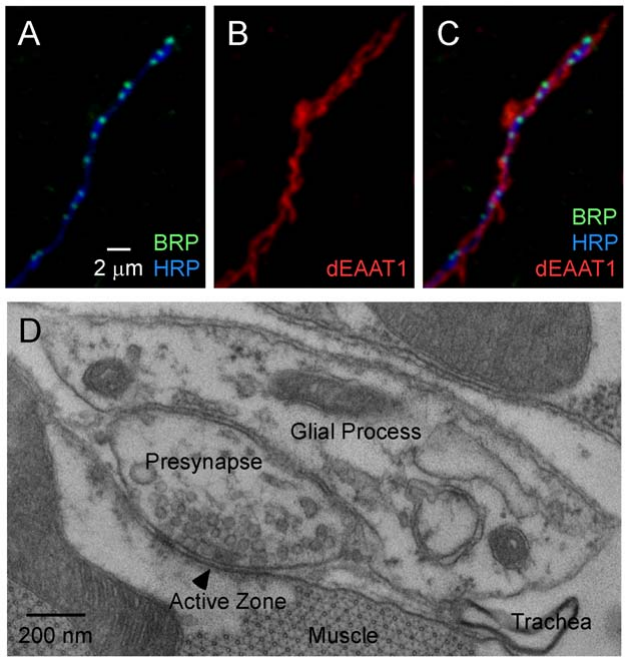
Tripartite morphology of DLM neuromuscular synapses. (**A-C**) Confocal immunofluorescence images of DLM neuromuscular synapses. (**A**) Anti-HRP labels the neuronal plasma membrane and anti-BRP labels presynaptic active zones. (**B**) Anti-dEAAT1 labels glial processes and (**C**) reveals their close association with axons and synapses. The postsynaptic muscle membrane is not labeled and appears dark. All images are maximum projections of three consecutive optical z-sections. (**D**) Ultrastructure of glia-synapse interactions at DLM neuromuscular synapses. The EM images shown here and in Figure S1 are from single sections containing active zones. Among 123 such images analyzed, perisynaptic glial processes were observed in 96 (78%). Because this percentage is based on single section profiles rather than three-dimensional reconstructions, it is expected to underestimate the percentage of synapses associated with glial processes. These findings are consistent with immunocytochemical observations indicating that most synapses are associated with perisynaptic glia. Glial processes could be identified on the basis of the distinctive intracellular morphology characterized by heterogeneous membrane structures (c.f. [22]).

As shown in Figure 1, presynaptic boutons of DLM neuromuscular synapses are enveloped by glial processes. This may be visualized through immunofluorescence of the endogenous glial-specific glutamate transporter, dEAAT1 (Figure 1A–C and [18]) and by ultrastructural analysis showing that glial processes cover the bouton surface which is not in contact with the muscle (Figure 1D). Note that regions of the presynaptic plasma membrane containing neurotransmitter release sites (active zones) are not directly covered by glia (Figure 1C, D). The images shown in Figure 1A–C are typical and essentially all synapses were found to be associated with glial processes. The TEM image in Figure 1D is a representative single section showing direct contact of a presynaptic bouton with a glial process. Two additional examples are provided in Figure S1. Encapsulation of synapses by glia has been described in detail for mammalian glutamatergic synapses such as the cerebellar climbing fiber (CF) to Purkinje Cell (PC) synapse [22]. Moreover, CF-PC and DLM neuromuscular synapses share additional morphological features including highly branched axons with small boutons containing only one or two active zones. These similarities may reflect overlap in the functional properties of these synapses and their roles in the nervous system. Both are high release probability synapses [16], [23] which can provide strong synaptic input to reliably drive their targets in an all-or-none fashion. Such reliable transmission is a hallmark of CF-PC synapses [24] and is expected to be critical for precise control of wing-beat frequency during *Drosophila* flight.

### Functional interactions of glial processes at DLM neuromuscular synapses: Regulation of extracellular glutamate and synaptically-induced glial calcium transients

Perisynaptic glia express glutamate transporters and play a key role in controlling extracellular glutamate at tripartite synapses. This has been examined both pharmacologically and through genetic disruption of the mouse glial glutamate transporters, GLAST (EAAT1) and GLT-1 (EAAT2) [25]–[31]. In *Drosophila*, a single high affinity glutamate transporter of the EAAT family, dEAAT1, is specifically expressed in glia [17], [20], [21]. Previous studies employed the GAL4-UAS system to achieve glial-specific RNAi-based knockdown of dEAAT1 and reported several resulting phenotypes including motor impairment in adult flies and neurodegeneration [18], [19]. Similarly, disruption of glial glutamate transporters in the mouse or rat led to glutamate excitotoxicity and neurodegeneration [31], [32].

**Figure 2:**
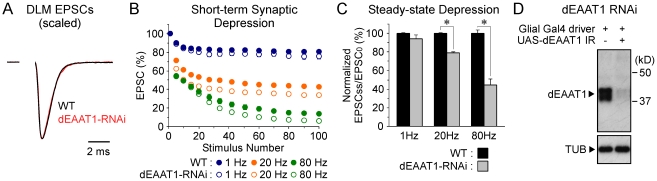
A Synaptic phenotype produced by glial-specific knockdown of dEAAT1. (**A–C**) Two-electrode voltage-clamp recordings of synaptic currents from DLM neuromuscular synapses. (**A**) Scaled and superimposed single EPSCs from wild-type (WT) and dEAAT1-RNAi synapses indicate similar EPSC waveforms. For the dEAAT1-RNAi condition, a glial-specific GAL4 driver (dEAAT1-GAL4) was used to express the UAS-dEAAT1-IR transgene. The initial EPSC amplitudes for WT and dEAAT1-RNAi conditions were 1.75±0.06 (n = 22) and 1.87±0.11 (n = 16), respectively, and were not significantly different (*p* = 0.32). (**B**) dEAAT1-RNAi synapses exhibit enhanced short-term depression during train stimulation at 1, 20 or 80 Hz. Peak EPSC amplitudes were normalized to the initial amplitude and plotted as a function of stimulus number. Error bars represent the S.E.M. (**C**) Enhancement of steady state short-term depression at dEAAT1 RNAi synapses at higher stimulation frequencies. Asterisks indicate statistical significance with respect to wild-type. (**D**) Confirmation of dEAAT1 knockdown by Western analysis of head homogenates. Glial-specific expression of the UAS-dEAAT1-IR transgene produced a marked reduction in dEAAT1 protein levels.

In the present study, knockdown of dEAAT1 in glia produced a clear defect in DLM neuromuscular synapse function. First of all, no change was observed in the waveform of the excitatory postsynaptic current (EPSC) elicited by a single stimulus (Figure 2A). The time for 50% decay of the EPSC (half-width) at wild-type and dEAAT1 RNAi synapses was 0.96±0.03 msec (n = 5) and 0.98±0.03 msec (n = 5), respectively, and were not significantly different (*p* = 0.73). These findings are consistent with previous observations in mouse knockout mutants for either GLT-1 or GLAST, which indicate that residual glial glutamate transport contributes to preserving the EPSC waveform [31], [33], [34]. This may also be the case for the present study although knockdown of dEAAT1 was found to be efficient (Figure 2D and [19]). Further analysis employing train stimulation indicated that glial-specific dEAAT1 knockdown produced clear enhancement of short-term synaptic depression with respect to wild type (Figure 2B,C) which was more prominent at higher stimulation frequencies. This phenotype may reflect accumulation of extracellular glutamate and resulting glutamate receptor desensitization. As described in the Discussion, a previous study has reported effects of glial-specific dEAAT1 knockdown on DLM neuromuscular synapse function [18].

**Figure 3:**
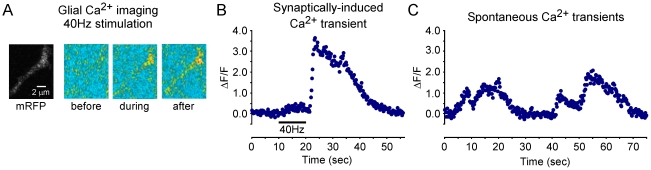
Synaptic activity-induced glial calcium transients. Fluorescence changes (ΔF/F) within glial processes expressing GCaMP3. (**A**) Example images of synaptic activity-dependent calcium transients in glial processes. Glial specific expression of the membrane-associated mCD8-mRFP protein provided a red fluorescent marker for glial processes. Pseudocolor images of GCaMP3 fluorescence before, during and after a 40 Hz stimulation train. Blue, yellow, red and white colors indicate progressively higher GCaMP fluorescence. (**B**) An increase in cytosolic calcium induced by DLM motor axon stimulation (40 Hz for 10 s). This response is representative of those observed in 18 out of 22 preparations (four preparations showed no response). Some variation was observed in the properties of activity-induced glial calcium transients with respect to their time of onset and response duration. Additional examples are provided in Figure S2. (**C**) Spontaneous calcium transients.

In addition to control of extracellular glutamate, a second functional interaction of glia at DLM neuromuscular synapses was revealed through calcium imaging in glial processes. Perisynaptic glia in several systems are known to exhibit transient cytosolic calcium increases either spontaneously or in response to synaptic stimulation [8], [35]–[42]. This form of signaling was also observed at DLM neuromuscular synapses (Figure 3) in experiments employing glial-specific expression of the genetically-encoded calcium indicator, GCaMP3 [43], [44]. Train stimulation of the DLM motor axon (40 Hz for 10 seconds) induced an increase in cytosolic calcium (Figure 3A,B). Although the response varied in its amplitude and time course, typically a modest increase in calcium was observed during the stimulation train followed by a larger calcium transient as described for cerebellar tripartite synapses [38], [40]. Additional examples of activity-induced glial calcium transients are shown in Figure S2. Finally, calcium oscillations also occurred spontaneously (Figure 3C) as reported for mammalian models [36], [41]. Calcium transients and propagating calcium waves are thought to participate in network interactions of glial processes [8], [45]–[47].

### Glial coupling of synapses with tracheal structures mediating gas exchange

**Figure 4:**
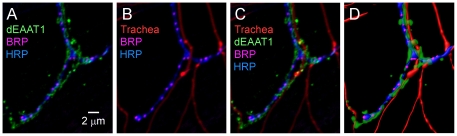
Glial coupling of synapses to trachea mediating gas exchange. (**A**) Confocal immunofluorescence images of DLM neuromuscular synapses as described in Figure 1. (**B**) Visualization of trachea by autofluorescence and (**C**) their association with glial processes and coupling to presynaptic boutons. (**D**) Three dimensional model of tripartite synapse interactions (generated with the Software package, Imaris).

Neurovascular coupling involves glial regulation of the vasculature in response to neural activity [48]–[50]. This process provides a mechanism to adapt the local circulation to changing demands of neuronal metabolism and respiration. Although cellular gas exchange in *Drosophila* is mediated by fine branches of tracheal (air) tubes rather than blood vessels, here we report that glial processes appear to couple trachea and presynaptic boutons at DLM neuromuscular synapses (Figure 4). We first observed that glial processes exhibit complex structures which often project beyond their axonal contacts. Upon further investigation, these structures were found to reflect interactions of glial processes with trachea. Although fine trachea and axons are often located in close proximity, these structures typically exhibit clear spatial separation and little direct contact (Figure 4B). Notably, glial processes occupy the gaps between tracheal and axonal structures (Figure 4C, D). This morphological coupling of axons and trachea by glial processes suggests perisynaptic glia may function in signaling related to gas exchange as described for glial-vascular coupling. Whether this analogous organization may reflect divergent or convergent evolution remains an interesting question. In either case, it may serve as a simple and attractive model for analysis of glial mechanisms regulating gas exchange and more generally glial network interactions involving tripartite synapses.

## Discussion

Although glia constitute the majority of cells in the nervous system, recognition of their critical importance in neural function is only beginning to approach that of neuronal mechanisms. Further progress will be facilitated by *in vivo* analysis of glial mechanisms in genetic model systems such as *Drosophila*. The present study advances one important aspect of this effort by establishing a *Drosophila* tripartite synapse model exhibiting morphological and functional properties similar to those of mammalian tripartite synapses.

Analysis of tripartite DLM neuromuscular synapses will benefit from classical and cell-type genetic approaches in *Drosophila*. For example, the availability of GAL4 driver lines for specific expression in the presynaptic, postsynaptic or glial compartments, in combination with UAS transgenic lines for RNAi-based knockdown of most *Drosophila* genes (c.f. [51]), should greatly facilitate molecular analysis of native tripartite synapses. Previous work in *Drosophila* has provided a thorough characterization of the glial glutamate transporter, dEAAT1 [17]–[21], and served as an important basis for aspects of the present study. Good agreement was found in general, however the previous study of DLM neuromuscular synapses reported a change in the time course of the excitatory postsynaptic potential (EPSP) following dEAAT1 knockdown in glia [18]. Smaller amplitude EPSPs, as produced by enhanced short-term depression in the dEAAT1 RNAi condition, were longer in duration. This likely reflects electrogenic contributions of the muscle membrane to the apparent EPSP time course as observed previously (c.f. [52]). In the current study, elimination of this effect through voltage clamp recording of EPSCs indicates no change in the EPSC waveform with respect to wild type (Figure 2A). Another significant technical difference is that DLM motor axons were severed in the current study to prevent endogenous activity and selectively assess the effects of exogenous axon stimulation. Finally, development of the tripartite DLM neuromuscular synapse model in *Drosophila* will be complementary to studies of central glial mechanisms contributing to higher order functions such as learning and memory [53], circadian rythyms [54] and courtship behavior [55].

The DLM neuromuscular synapse exhibits a unique combination of features among tripartite synapse models. The overlap of its morphological and functional properties with those of mammalian glutamatergic synapses, in combination with classical and cell-type specific genetic approaches available in *Drosophila*, enhances its value as a general model. The simplicity and accessibility of the DLM neuromuscular synapse provide additional advantages which are shared with tripartite mouse neuromuscular synapses, along with morphological similarities and synaptically-induced glial calcium transients [41], [56]. However, because the DLM neuromuscular synapse is glutamatergic rather than cholinergic, it may serve as an accessible model for analysis of glial interactions at glutamatergic synapses. In light of its unique properties, genetic analysis of the DLM neuromuscular synapse may further define conserved mechanisms of tripartite synapse function in neurological health and disease.

## Materials and Methods

### *Drosophila* Strains

Glial specific transgene expression was achieved using the GAL4-UAS system [57]. The glial-specific GAL4 driver line, dEAAT1-GAL4, and the dEAAT1 RNAi transgenic line, UAS-dEAAT1-IR-II [19], were generously provided by Dr. Serge Birman (Developmental Biology Institute of Marseille, France). The glial-specific Repo-GAL4 enhancer trap line [58] and the UAS-mCD8-mRFP transgenic line were obtained from the Bloomington Stock Center. The UAS-GCaMP3 transgenic line [44] was generously provided by Dr. Loren Looger (Janelia Farm Research Campus, HHMI). Wild-type flies were Canton-S. Stocks and crosses were cultured on a conventional cornmeal-molasses-yeast medium. Flies were reared at 20-22°C or the indicated temperature.

### Immunocytochemistry and Confocal Microscopy

Immunocytochemistry was performed essentially as described previously [59]. These studies employed the following primary antibodies: Rabbit anti-dEAAT1 (1∶2500) [provided by Dr. Serge Birman (Developmental Biology Institute of Marseille, France)]; mAb nc82 anti-BRP (BRUCHPILOT) (1∶50) [Dr. Erich Buchner (Universitaet Wuerzburg, Germany)]; Cy5-conjugated rabbit anti-HRP (1∶200) (Jackson Immunoresearch Laboratories, West Grove, PA). Secondary antibodies included Alexa Fluor 488-conjugated anti-rabbit IgG (1∶200) and Alexa Fluor 568-conjugated anti-mouse IgG (1∶200) (Invitrogen, Carlsbad, CA). Trachea were visualized by autofluorescence with excitation at 405 nm. Imaging was performed using an Olympus FV1000 confocal microscope (Olympus Optical, Tokyo, Japan) with a PlanApo 60× 1.4 numerical aperture oil objective (Olympus Optical) and a z-step size of 0.2 µm. Images were acquired and processed with Fluoview software (Olympus Optical). Three dimensional representations of confocal image stacks were generated using the software package, Imaris (Bitplane Scientific Software, St. Paul, MN). After the confocal image stack was loaded into Imaris, a “surface object” was created for each channel to generate the 3-D model. The intensity threshold for each surface object was adjusted to best match the original image. The opacity of surface structures was adjusted to permit visualization of underlying structures.

### Synaptic Electrophysiology

Excitatory postsynaptic currents (EPSCs) were recorded at dorsal longitudinal flight muscle (DLM) neuromuscular synapses of 3 to 5 day-old adults reared at 20°C. These experiments were performed as described previously [16]. Briefly, two-electrode voltage clamp was performed with a TEV-200 amplifier (Dagan Corporation, Minneapolis, MN). The recording solution consisted of (in mM): 128 NaCl, 2 KCl, 4 MgCl_2_, 1.8 CaCl_2_, 5 HEPES, and 36 sucrose. The pH was adjusted to 7.0 using NaOH. Temperature control was achieved using a TC-202A temperature controller and PDMI-2 microincubator (Harvard Apparatus, Holliston, MA). EPSCs were recorded at a holding potential of −20 mV. Data were acquired using Pulse software (Heka Electronik, Lambrecht, Germany) and an ITC-16 laboratory interface (Instrutech, Great Neck, NY). Stimulation of DLM motor axons was achieved with a Master-8 Stimulator (A.M.P.I., Jerusalem, Israel). Synaptic currents were low-pass filtered at 5 kHz and acquired at 25 kHz. Measurements of synaptic current amplitudes and half-width measurements of the EPSC decay phase were carried out in the Mini Analysis Program (Synaptosoft, Decatur, GA). For half-width measurements, minimal stimulation of the motor axon was utilized to ensure optimal preservation of the EPSC waveform.

### Transmission Electron Microscopy

TEM was performed on a JEOL JEM 1200 EXII microscope housed at the Penn State University Electron Microscopy Facility. These studies employed conventional methods, essentially as described [60], [61]. Briefly, initial fixation was carried out by exposing the preparation to primary fixative composed of 2.5% paraformaldehyde and 1.5% glutaraldehyde in recording solution (see Synaptic Electrophysiology). Samples remained in primary fixative at room temperature for 1–3 hr and then overnight at 4°C. Fixed samples were processed in 2% aqueous osmium tetroxide and then in 2% aqueous uranyl acetate, dehydrated using an ethanol series, and embedded in Spurr resin (EM Sciences, Fort Washington, PA). Thin sections (∼60 nm) were cut on an ultramicrotome and stained with uranyl acetate/lead citrate before imaging.

### Glial Calcium Imaging

For glial-specific expression of the genetically-encoded calcium indicator, GCaMP3, flies carrying the glial-specific repo-GAL4 driver [58] and UAS-GCaMP3 [44] transgenes were reared at 25°C. GCaMP3 was excited at 488 nm. Time-lapse imaging was performed on an Olympus FV1000 confocal microscope (Olympus Optical, Tokyo, Japan) with a 60× 1.0 numerical aperture water-immersion objective (Olympus Optical). Images were processed using Metavue software (Molecular Devices, Sunnyvale, CA).

### Data Analysis

Microsoft (Seattle, WA) Excel was utilized to analyze numerical data and generate graphs. All data values are presented as mean ± SEM. Statistical significance was determined using the two-tailed Student's t test and significance was assigned to comparisons for which *p*≤0.05.

## Supporting Information

Figure S1 **Examples of DLM tripartite synapse ultrastructure as described in** **Figure 1D** **.** Panels A and B show synaptic profiles in which segments of glial processes may be seen coupling synapses and trachea.
(TIF)

Figure S2 **Examples of synaptic activity-induced glial calcium transients as described in** **Figure 3** **.** Panels A and B reflect variation in glial calcium transients, including an example in which a small amplitude transient is observed during the stimulation train (**A**) and one in which the response fluctuates to produce a second calcium transient (**B**).
(TIF)
