# Supplementary figures and images for: A Tripartite Synapse Model in Drosophila
Source: PLoS One. 2011 Feb 16;6(2):e17131. doi: 10.1371/journal.pone.0017131 (PMC3040228; doi:10.1371/journal.pone.0017131)

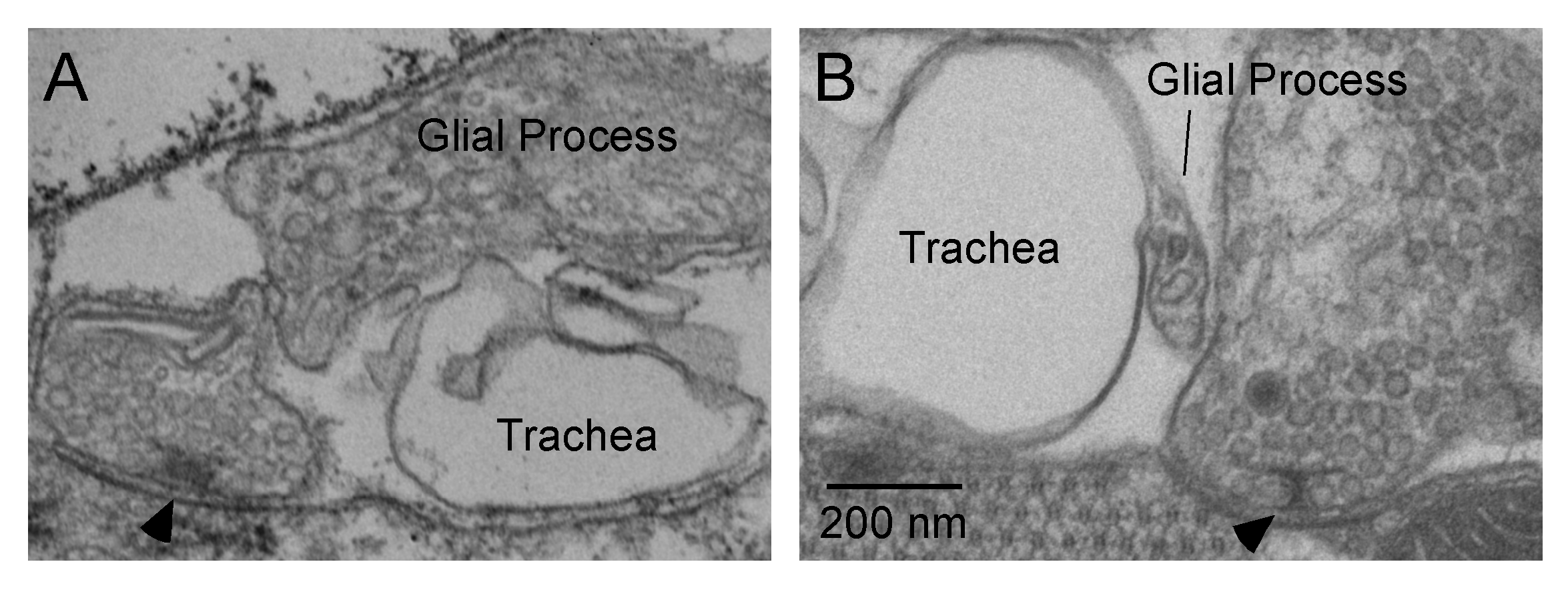

Supplement: Figure S1 — Examples of DLM tripartite synapse ultrastructure as described in Figure 1D . Panels A and B show synaptic profiles in which segments of glial processes may be seen coupling synapses and trachea. (TIF) [file pone.0017131.s001.tif]

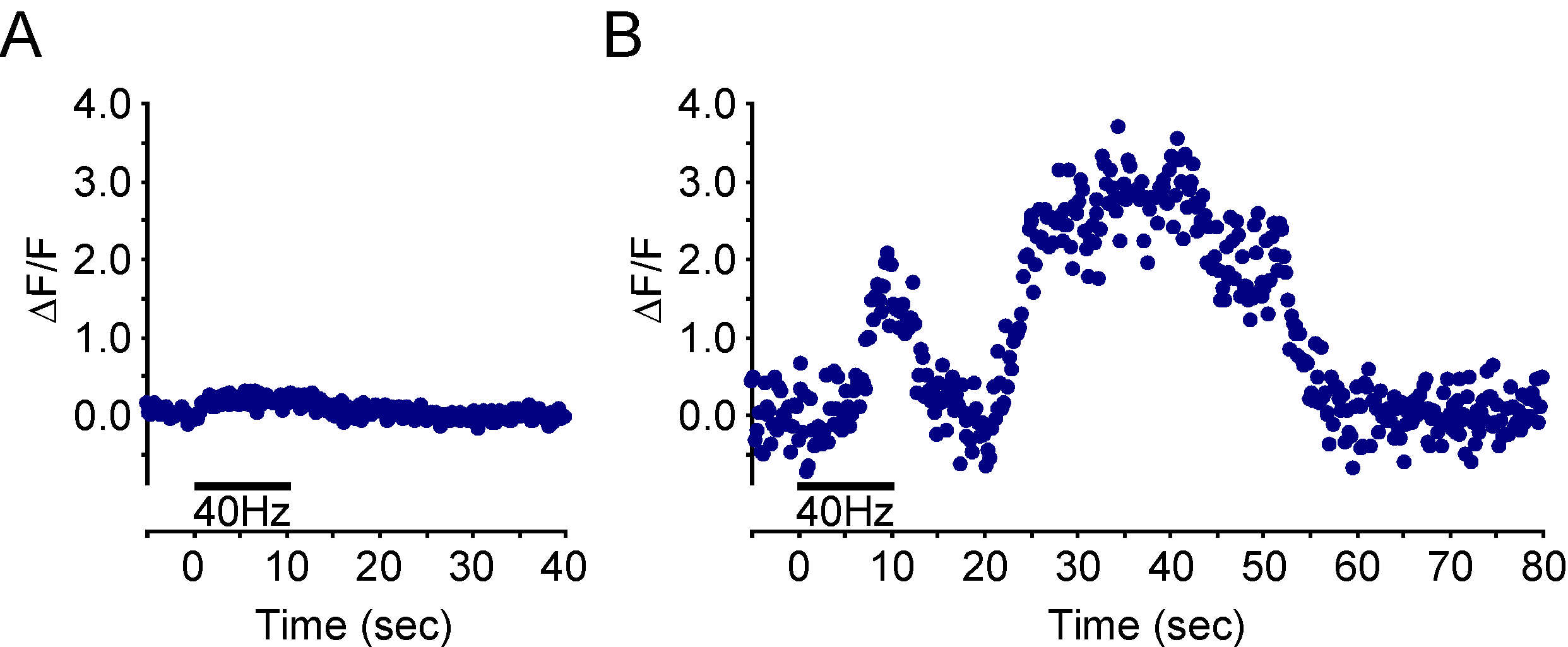

Supplement: Figure S2 — Examples of synaptic activity-induced glial calcium transients as described in Figure 3 . Panels A and B reflect variation in glial calcium transients, including an example in which a small amplitude transient is observed during the stimulation train (A) and one in which the response fluctuates to produce a second calcium transient (B). (TIF) [file pone.0017131.s002.tif]
